# Phosphorus and potentially toxic elements in stream waters, sediments, mine tailings, and pines across Lake Anna watershed, VA, USA, and biochar-lime remediation

**DOI:** 10.1007/s11356-025-37328-w

**Published:** 2026-01-13

**Authors:** Robert T. Kohlhaas, Sophia E. Schroeder, Justin B. Richardson

**Affiliations:** Department of Environmental Sciences, University of Virginia, 291 McCormick Rd, Charlottesville, VA 22904 USA

**Keywords:** Watershed biogeochemistry, River water, Acid mine drainage, Heavy metals, Nutrients

## Abstract

Lake Anna is an important ecological and recreational body of water in Virginia but struggles with stream water and sediment quality impairments due to historical and modern anthropogenic activities. The overarching goal was to quantify potentially toxic elements (PTEs) (As, Cr, Cu, Pb, Ni, and Zn) and P pollution across nine subwatersheds and the outflow of Lake Anna to evaluate the role of exposed mine tailings, agriculture, and physicochemical properties on sourcing and transport. Phosphorus enrichment in stream waters and sediments was associated with DOM and Fe, but not agricultural land cover. Suspended sediment total annual P export (191 Mg/year) was greater than dissolved P export (0.7 Mg/year), but < 56% of the P was retained within Lake Anna. Stream water, suspended sediment, and bottom sediment Cr, Cu, Pb, and Zn concentrations at Contrary Creek subwatershed (CC) exceeded ecologically hazardous concentrations and was a major PTE source for the reservoir. Comparing total annual exports, Lake Anna was a net accumulator of PTEs and P. Exposed mine tailings at CC had ecologically hazardous concentrations of As, Pb, and Cu, but pine tree needle PTEs were not elevated, demonstrating limited bioavailability. Lastly, our column experiment using exposed mine tailings found an application rate of 9.2 tonnes/ha of lime and biochar could decrease the leaching of Pb and Zn and increase leachate pH but could not significantly reduce As or Cu. Additional research of subsurface transport pathways and mobility of legacy sediments is warranted to immobilize PTE transport.

## Introduction

Degradation of water and sediment quality from pollutants continues to be an issue across the eastern United States due to historical and modern anthropogenic activities such as mining (e.g., Acharya and Kharel 2020) and industrial agriculture (Paudel and Crago 2021). Across the USA, organic pollutants such as pesticides are common; 221 pesticides were found in 74 river sites across the conterminous USA by Stackpoole et al. (2021). Although significantly reduced, excessive nitrogen and phosphorus degrade streams across the USA; Paudel and Crago (2021) showed a ratio of 10% increase in application leads to ~ 1.5% increase in N and P concentration across a watershed. Further, mining in geologic formations susceptible to acid mine drainage (AMD) can lead to release of potentially toxic elements (PTEs) such as As, Cd, Cu, Mn, Pb, and Zn (Kimball et al. 1995; Acharya and Kharel 2020). Additionally, metal pollutants in streams can occur from historical and modern sources such as industrial activities, such as manufacturing and municipal activities (e.g.,Deblonde et al. 2011; Butler et al. 2023).

Water quality is of particular importance for the Mid-Atlantic region of the USA due to the shared export to the Chesapeake Bay. The Chesapeake Bay is a key natural and economic resource; it is the largest tidal estuary on the Atlantic coast and one of the largest estuaries in the world (Morgan and Owens 2001). The Chesapeake Bay Foundation estimates that the commercial seafood industry in Maryland and Virginia have combined sales exceeding $2.8 billion US dollars and 20,000 jobs (National Marine Fisheries Service 2014). Unfortunately, excessive nutrient and metal contamination has been widely documented throughout the Chesapeake Bay watershed. According to Kleinman et al. (2019), 23 of the 66 monitoring stations within the Chesapeake Bay main tributaries have observed increasing total P loads from 2007 to 2016 and that legacy P pollution within sediments can be remobilized. Comeleo et al. (1996) demonstrated that elevated concentrations of PTEs such as As, Cr, Cu, Ni, Pb, and Zn across parts of the Chesapeake Bay posed direct ecological hazards to sediment filtering organisms.

Here, we focus on Lake Anna, which is a reservoir located in northcentral Virginia approximately 100 km southwest of Washington DC. Lake Anna was created in 1972 by Virginia Electric and Power Company through damming a portion of the North Anna River (Odhiambo et al. 2013). Lake Anna serves multiple roles as a recreational boating area, a popular freshwater fishing center for the region (32 different fish species including striped bass; see Odenkirk 2016), and important habitat for waterfowl, reptiles, and mammals. However, its principal role is to serve as a cooling water source for the North Anna Nuclear Power Station (US Nuclear Regulatory Commission 2007).

Water and sediment quality are impaired across Lake Anna from historical and modern anthropogenic activities (Odhiambo et al. 2013). The Lake Anna watershed is located in the Gold-Pyrite Belt mining district that stretches across central Virginia, and the region has been home to over 20 mineral mines (Dagenhart 1980; Pavlides et al. 1982). Mining activities for iron, copper, and sulfur can be traced back to the mid nineteenth century, predating the Civil War. Gold has been a primary output but other metals and minerals associated with gold veins, such as pyrite, Cu, Zn, and Pb, were also mined (Wood 1973; Dagenhart 1980). Commercial production of Au continued until 1947, when the last gold mine in the state ceased operations (Spears and Upchurch 1997). Mining for other metals such as Cu and Zn in Louisa County continued until the 1970 s via surface pits and underground mine shafts (Sweet et al. 1989). Many of the mines have been purportedly reclaimed for both AMD and metal pollution. As noted by Katz (1961), Wood (1973), and Dagenhart 1980, sulfide minerals were primarily pyrite (FeS_2_), pyrrhotite (FeS), sphalerite (ZnS), chalcophyrite (CuFeS_2_), and galena (PbS). Modern active AMD remediation techniques focused on oxygen removal while passive AMD remediation aim to capture released acids of metals (Rambabu et al. 2020; Zhang et al. 2023). Previous efforts for reclamation in the Lake Anna watershed in the late 1970 s used straw, sludge, and lime to decrease AMD and potentially toxic elements (PTEs). Straw and other similar agricultural byproducts have been shown to add soil carbon, increase sulfur reducing bacteria, and adsorb metals but may not reduce the bioavailability or mobility of PTEs (e.g., Sarathchandra et al. 2024). These efforts within the Lake Anna mines have yielded mixed results, as stream water Pb, Cu, and Zn concentrations have not significantly decreased from remediation implementated between 1976 and 1980 (Hinkle 1982). Additional reclamation is needed with the potential use of liming and biochar as passive treatments to improve pH and adsorb metals (Niazi et al. 2018; Tomczyk et al. 2019; Acharya and Kharel 2020; Sharma et al. 2022; Sun et al. 2022). Biochar by itself has not been shown to be effective in adsorption of PTEs (see Acharya and Kharel 2020), but the co-application with a liming agent could serve as a sustainable passive treatment (Wibowo et al., 2023). Due to the mixed success in remediation for the mines in Lake Anna watershed, continued evaluation of the long-term efficacy of these remediation projects and considering additional measures is essential as recreation and sport fishing use increases.

Agriculture and human developments also serve as sources of nutrient pollution to Lake Anna, primarily through releases of N and P. For the two main counties in the Lake Anna Watershed – Spotsylvania and Louisa Counties –, there were 790 farms with 48% of the farms engaged in row crops and 52% engaged in livestock and poultry in 2021 (USDA National Agricultural Statistics Service 2022). Common livestock and poultry operations include horses, turkeys, chicken, sheep, beef cattle, and goats while common row crops include grains, vegetables, fruits, and hay pastures in 2021 (USDA National Agricultural Statistics Service 2022). With greater peri-urban development stemming from the expansion of the Washington DC metropolitan area, human home developments are increasing in the Lake Anna watershed. Although P loading from these non-point and point sources act have decreased between 1985 and 2019, these important sources of P to stream waters still lead to algal blooms and eutrophication (see Sabo et al. 2022). Dissolved forms of P (primarily as inorganic forms of phosphate, e.g., House 2003; Kao et al. 2022) and suspended sediments (Mallin and Cahoon 2020; Rose & Karwan, 2021) are the dominant forms of P transported from soils and riparian areas to streams (Fox et al. 2016). With historical legacy P potentially buried in bottom sediments and dissolved and suspended particulate P from non-point sources, multi-medium assessments are needed to determine current P export rates and their relationships to land use to capture the status of P pollution from the tributaries across Lake Anna.

The overarching goal of this study was to quantify potentially toxic elements (PTEs) such as As, Cr, Cu, Pb, Ni, and Zn and nutrient (here only total P) pollution in diverse tributaries of Lake Anna from former mines and agricultural lands as well as processes affecting their transport (i.e., acidity, organic matter, particulates). We also examined if exposed mine tailings releasing PTEs can be remediated with low-cost materials biochar and lime. For our first objective, we evaluated stream water, suspended sediments, and bottom sediments to determine nutrient and trace element loading rates and mechanism into Lake Anna. For our second objective, we evaluated if exposed mine tailings had mobile phases of trace elements and elevated concentrations of toxic elements. In our final objective, we conducted a laboratory experiment to evaluate if biochar and lime could substantially diminish the solubility and leaching of toxic elements from the mine wastes. Quantifying current fluxes and mechanisms of pollution transport into Lake Anna is essential for improving water and sediment quality entering the reservoir.

## Methods and materials

### Description of Lake Anna

Lake Anna is a human-made reservoir formed by the North Anna Dam in Louisa County, VA. The lake was created in 1972 to serve as a cooling pond for the North Anna Nuclear Generating Station, which went into commercial operation in 1978. Lake Anna is physiographically in the Virginia piedmont characterized by rolling hills, deciduous forests, farmland, and minor urban towns and villages. The Virginia piedmont has a humid subtropical climate with a mean annual temperature of 15 °C and mean annual precipitation of 1190 mm year^−1^. Lake Anna covers an area of 53 km^2^ with a drainage basin of 885.78 km^2^ and is a recreational reservoir and non-potable water body. Lake Anna is in the North Anna River watershed and is part of the greater York River watershed. The piedmont region of Virginia has bedrock composed of late-Proterozoic and Paleozoic igneous and metamorphic rocks but also includes several basins of sedimentary rock from the Triassic period (Bailey et al. 2014). According to the National Land Cover Database 2021 analysis (USGS 2024), Lake Anna watershed is mostly forested land (56%), with abundant agricultural land (20%) and moderate amounts of shrub and grassland (8%), urban development (8%), and open water (6%) (see Table 1).

**Table 1:** Description of nine subwatersheds and Lake Anna outflow properties and land covers based upon NLCD 2021 dataset (Homer et al., 2020). Forest includes deciduous, evergreen, and mixed forests. Agriculture (Agri.) includes cultivated crops and pastures. Developed (Dev.) includes low intensity, medium intensity, and high intensity development but not open space

Watershed	Watershed code	Watershed area	Forest	Agri.	Dev.	Pasture/grass
		km^2^	%	%	%	%
Contrary Creek	CC	17.97	80	6	8	12
Freshwater Creek	FW	23.08	69	5	7	13
Goldmine Creek	GM	57.24	59	21	6	10
North Anna River	NA	133.38	64	18	7	8
Terry’s Run	TR	69.67	60	27	7	5
Pigeon Run	PR	10.93	69	2	4	19
Pleasant Run	PLR	8.13	63	27	5	3
Foremost Run	FR	10.02	50	35	4	8
Pamunkey Creek	PC	133.48	49	35	9	5
North Anna River Outflow	NAO	885.78	56	20	8	8

### Description of subwatersheds studied

**Fig. 1:**
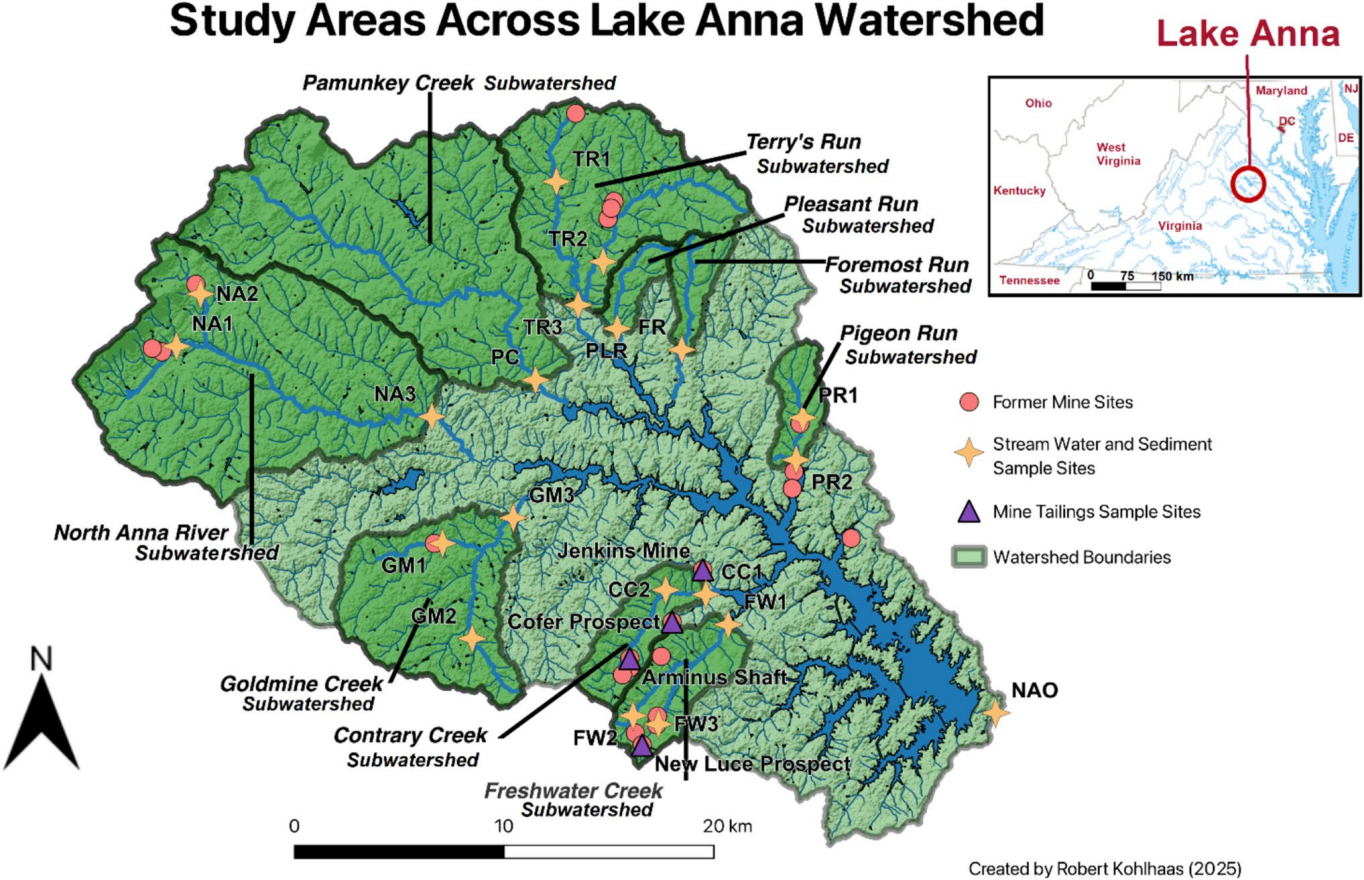
Map of the Lake Anna watershed and subwatersheds within study areas. Former mining operations are indicated with red circles, location water mines studied are indicated with purple triangles, location of sediment and stream water sampling are indicated with yellow star, and their subwatersheds studied are labeled and have darker green fill

We studied nine subwatersheds and the outflow of the main watershed to evaluate the effects of agriculture and historical mining as sources of elements degrading water quality of Lake Anna. The abundance of agricultural lands (Table 1) and former mine sites (Fig. 1) varies between sub-watersheds of Lake Anna, providing reasoning for the varying abundances of PTEs and P. This variation in land use allows for assessing their impacts on nine tributaries and twenty total stream sites across the entire basin, including the North Anna River outflow (Fig. 1).

Contrary Creek (CC) subwatershed was one of our main focus due to its historical and present-day acid mine drainage (AMD) issues. Within the area are three former mines: the Arminus Shaft (pyrite, Zn, Cu), the Cooper mine (Au), and the Walton mine (Au). Both stream water and sediment sample sites (CC1 and CC2) are downstream from the former mine sites, while mine tailing sampling occurred at the Arminus Shaft site.

The Freshwater Creek (FW) subwatershed is also on the central-south side of Lake Anna. The subwatershed includes three stream water and sediment sampling locations: the Freshwater Creek stem (FW1), Freshwater Creek tributary (FW2), and an unnamed tributary (FW3). The study area encompasses drainage from the town of Mineral, VA, and includes five former mine sites, of which only two are named: the New Luce Prospect (gold) and the Contrary Creek Mine (pyrite). The other unnamed mine sites include two former Au mines and one former Fe mine. The New Luce Prospect and the unnamed Fe mine are upstream from the unnamed tributary sample site.

The Goldmine Creek (GM) subwatershed study area inputs from the south of the northwest end of Lake Anna. The subwatershed includes three sampling sites: White Creek (GM1), Goldmine Creek tributary (GM2), and Goldmine Creek stem (GM3). One unnamed former mine is present just upstream from the White Creek sampling location. Most of the town of Louisa, VA, is included in the Goldmine Creek drainage area.

The North Anna River (NA) subwatershed study area is located on the northwest side of Lake Anna. The subwatershed includes three sample sites: Mountain Run (NA1), Madison Run (NA2), and the North Anna River (NA3). Upstream from Mountain Run are two former limestone quarries, Cowherd Quarry and one unnamed. Upstream from Madison Run is a former marble quarry. The North Anna River site NA3 is near the densest urban land as it encompasses Gordonsville, VA.

The Terry’s Run (TR) subwatershed study area is on the north side of Lake Anna. The area is mostly forested but has a relatively large amount of agricultural land. Three sampling sites are included in the study area: Riga Run (TR1), Terry’s Run tributary (TR2), and Terry’s Run stem (TR3). An unnamed clay mine is located upstream from the Riga Run sample point, and the Young Prospect site and two unnamed mines—all three, former Au mine sites—are upstream from the Terry’s Run tributary sample point.

The Pigeon Run (PR) subwatershed is on the central northside of Lake Anna, including a portion of Lake Anna State Park. Forested land dominates the watershed, though it also contains a relatively high grassland proportion, and the lowest percentage of agricultural land at 2%. Two sampling sites are found in the study area, both on the singular stream of Pigeon Run. The first of these two sites (PR1) is located just upstream from the Goodwyn Mine, a former Au mining site, while the second site (PR2) is downstream from the former mine, located just before Pigeon Run flows into the Lake. The purpose of these site locations was to gain insight on any key differences between ecosystem health upstream and downstream from the Goodwyn Mine.

The Pleasant Run (PLR) is the smallest subwatershed, located on the north side of Lake Anna, bordering the right side of the Terry’s Run study area. The watershed is mostly forested land with a large amount of agricultural land and very little shrub and grassland. Only one sampling point is found in the study area, as there are no substantial tributaries to Pleasant Run. No former mine sites are found in this study area.

The Foremost Run (FR) subwatershed is on the north side of Lake Anna. The study area has one of the lower proportions of forested cover (50%) and one of the highest proportions of agricultural land (35%). This location was chosen due to its density of and proximity to agricultural lands, with the actual sample point being located in proximity to grazing cow pastures.

The Pamunkey Creek (PC) subwatershed area is one of the largest subwatersheds, located on the north side of Lake Anna. This study area has the lowest proportion of forested land cover (49%) and the highest amount of agricultural land (35%) of any of the other watersheds studied. There are moderate proportions of developed land (9%) and shrub and grassland (5%) found in the Pamunkey Creek study area as well. The single sampling point is located in the middle of a cultivated pasture.

The North Anna Outflow (NAO) serves as the output from the entire 885.78 km^2^ area of the Lake Anna watershed, capturing the discharge into the North Anna River that eventually drains to the Chesapeake Bay via the York River. The singular sample point is located just downstream from the Lake Anna Dam and provides insight on export of elements out of the reservoir. It is important to note that there are three former mine sites that are included in the Lake Anna Watershed and North Anna Outflow study area that were not captured by the other study areas: the Edenton mine (mica), the Jenkins Mine (pyrite), and the Cofer Prospect (Zn). Mine tailing samples were taken from both the Jenkins Mine and the Cofer Prospect. There are point sources of elements from waste water treatment facilities, industrial activities, and coolant water from the North Anna Nuclear Generating Station.

### Stream water and sediment sampling

#### Stream water sampling and USGS gauge data

Stream water samples were taken in June and July (Summer) and end of August (Fall) December 2024 and January 2025 (Winter) and May 2024 and February 2025 (Spring). A total of 148 stream water samples were collected and analyzed. All samples were collected using a telescopic beaker rod and stored in 500-mL polyethylene bottles. Each bottle was rinsed with the respective stream water before the sample for analysis was collected, filling with no headspace. Samples were taken from the center of each stream, but in the cases of larger streams, water from about 2 m from the stream bank was collected. Water was also sampled in locations of steady flow without turbulence. Directly after sampling, waters were tested for their pH and oxidation–reduction potential (ORP) using a Fisher Accumet AB315 pH/mV probe and meter. Samples were then filtered, using 0.45-μm Whatman nitrocellulose membrane filters, and acidified using 1 mL of 35% HCl acid. Prior to elemental analysis, a subsample was diluted 1:1 with 2% HNO_3_ solution.

Stream discharge data for March 2024 to March 2025 was gathered from USGS stream gauges, which are operational on 3 of streams sampled: Pamunkey Creek (PC = station 01670200), North Anna River (NA3 = station 016701405), and North Anna Outflow (NAO = station 01670400). Stream gauge data indicated a relatively dry summer, with a maximum discharge event occurring in late July of 2024. The winter months 2024–2025 experienced a greater than average discharge at all three gauges compared to the summer of 2024. Large discharge events occurred intermittently from mid-January through mid-February of 2025 from several snowmelt events. Samples taken in February were during one of these snowmelt events.

#### Stream bottom sediments

Bottom sediment collection occurred at 20 locations distributed across the nine subwatersheds over the spring and summer of 2024. Site locations were determined by either their proximity to former mine sites or agricultural lands within the nine subwatersheds. Using a hand auger and a trowel, 3 to 6 bottom sediment samples were taken at each site with one to three sites per subwatershed. A total of 156 bottom sediment samples were analyzed in this study. Samples were taken at depths were generally 5 to 10 cm but no greater than 15 cm to capture sediments most likely mobilized during high flow events and readily available to the surface. Bottom sediment samples were oven-dried at 50 °C to a constant mass. Samples were sieved to particle sizes ≤ 2 mm. Bottom sediment pH was completed with a CaCl_2_ solution, where ~ 5 g of sample and ~ 12.5 g of aqueous CaCl_2_ (1.47 g L^−1^) were combined and shaken in 50-mL tubes. After settling for at least 2 h, sediment solutions were measured for pH using a Fisher Accumet AB315 pH/mV probe and meter. Sediment particle analysis was done by mixing 30–40 g of a sample with sodium hexametaphosphate (HMP) solution (50 g L^−1^) in a 250-mL beaker. After letting the slurry rest overnight to ensure sediment deflocculation, it was stirred up and poured into a 1000-mL graduated cylinder and diluted with tap water. The solution in the graduated cylinder was mixed and a hydrometer was used to measure sample density after 60 s and after 1.5 h. Hydrometer measurements were rounded to the nearest 0.5 g L^−1^, and a blank sample was observed to account for any discrepancies with the tap water. Organic matter content of sediment was determined by loss-on-ignition (LOI). In ceramic crucibles, ~ 10 g of sediment was weighed out and combusted within a muffle furnace set to 550 °C for at least 8 h, and sample masses were reweighed. The mass difference before and after ignition was used to determine the percent of organic matter (%OM) for each sediment sample. Lastly, a subsample of the bottom sediments was digested for elemental analysis. In brief, 2.0 g of sediment was weighed out and digested with 5 mL of reverse aqua regia (9:1 70% HNO_3_ to 35% HCl) following EPA Method 3050B (see Peña-Icart et al. 2011). Every batch of 25 suspended sediments included a digestion blank and 0.50-g sample of the standard reference material (SRM) of Montana Soil 2710a from the National Institute of Standards and Technology.

#### Suspended sediments

Suspended sediments were collected from the stream water during the 0.45-μm Whatman membrane filtration. Filters were weighed before and after the filtration process. Each batch of filters was dried and weighed out with a microbalance with an accuracy of 0.1 mg. Suspended sediment was placed in 50-mL tubes and digested with 5 mL of reverse aqua regia (9:1 70% HNO_3_ to 35% HCl) following EPA Method 3050B. Every batch of 25 suspended sediments included a blank membrane filter and a 0.05-g sample of Montana Soil 2710a SRM. The digested samples were heated using a hot plate with tube holders at 90 °C for 45 min and then diluted using deionized (DI) water to 50 mL. Before instrument analysis, the diluted digest was diluted to 15 mL with deionized water.

#### Dissolved and suspended sediment PTE and P watershed export

We utilized stream water and suspended PTE and P masses and USGS river discharge data from January 2024 to March 2025 to estimate watershed scale export rates from each subwatersheds and the entire Lake Anna watershed at the reservoir outlet. First, daily discharge rates were summed to determine total monthly discharge using USGS discharge stations (Pamunkey Creek Station 01670200; North Anna River station 016701405; South Anna River 01672500) and estimate for the ungauged watersheds using linear regression (see Richardson 2020). Next, the monthly water and suspended sediment discharge and the PTE and P concentrations were multiplied to determine export mass of elements per month. For months when samples were not collected, we used the closest temporal sample within the same season. The monthly dissolved river water and suspended sediment were summed to estimate a total annual export rate. Normalized dissolved and suspended sediment watershed exports were calculated to compare across watersheds by dividing the total annual export rate by total watershed area. These methods underestimate export rates of dissolved and suspended sediments during large storms events or other unknown stochastic large fluxes (e.g., high volume wastewater treatment release or post-application fertilizer run-off spike) but provide an empirical approximation across our study subwatersheds.

### Mines studied and collection of mine tailings

Due to the known AMD and elevated concentrations of metals in Contrary Creek, we conducted a study of four exposed mine tailing piles as potential sources. We utilized aerial photography and orthosatellite imagery to identify persistent bare patches devoid of vegetation from 1980 to 2020. We then leveraged the Virginia Department of Energy interactive map of abandoned mineral mined land and Virginia Division of Mineral Resources publications (Sweet and Trimble 1983) to confirm the legacy mines. We focused on four mines: Cofer Prospect, Jenkins mine, Arminus Shaft, and New Luce Prospect. At each mine, we collected mine tailing samples and identified three locations at least 15 m away from the tailing piles as reference sites. Mine tailings and reference soils were collected on-site in March and July 2024 by auger at three locations at three different depths of 5 cm, 10 cm, and 15 cm depth, at each site. Pine needles from surrounding pine trees present at the sites, Virginia pine and loblolly pine, were collected from ten different pine trees per mine tailing and reference sites. Reference samples were taken within 0.2–0.8 km of each site using the same sampling procedure previously described. All samples were stored in polyethylene bags.

Cofer Prospect (38° 3′ 5.54″ N, 77° 52′ 19.50″ W) was a shaft mine that operated in the 1950s, 5.50 km northeast of Mineral, Louisa County, VA. Although never operating as a commercial mine, the site was explored for pyrite and two vertical shafts were created. The last known date of mining activity was 1975. The mine tailing pile was mostly devoid of vegetation, except for some sparse patches of grass and a young forest of loblolly pines (*Pinus taeda*) and Virginia pines (*Pinus virginiana*). Records from Virginia Energy recommended reclamation of the mine tailings which included straw, lime, and sludge applications in the 1970s.

Arminus Shaft (38° 2′ 13.28″ N, 77° 54′ 2.30″ W) is located ~ 3.25 km north-east of Mineral, VA, and was historically a pyrite, zinc, gold, and copper mine. The four mine shafts were operational from 1865 to 1921. The mine tailings are surrounded by planted loblolly pine; however, the site itself possessed little vegetation, and loblolly pines on the edge of the tailings were sampled. Overland drainage from Arminus Shaft drains directly to Contrary Creek. Virginia Energy records advise cleanup and note previous semi-successful reclamation projects of 12 bales of straw, 80 tonnes of lime, crushed rock, fertilizers, and sludge applications in the 1970s.

Jenkins mine (38° 3′ 43.88″ N, 77° 51′ 35.03″ W), also known as the Allah Cooper Mine, is ~ 0.50 km from the mouth of Contrary Creek; it was first discovered in the 1830s with production beginning in 1835. The underground shaft mine primarily extracted native gold, though production was small. Notably, the mine tailings are located along the bank of Contrary Creek, which was been built from dredged mine tailings from the creek onto land. Reclamation of these dredged mine tailings has not occurred. Reference soils were collected upstream from the dredged tailing pile, while the tailings were studied directly, and a few Virginia pines were sampled.

Located ~ 1.50 km south-east of the town Mineral, New Luce Prospect (37° 59′ 48.192″ N, 77° 53′ 54.96″ W) was a prospective gold mine, with both underground and surface extraction attempts. A partial cap of sawmill waste was covering the site and lime had been applied as reclamation. The tailing samples were taken from the beneath the sawmill wastes and loblolly pine needles collected at the edge of the pile. Reference soils were collected 0.2 km away still within planted loblolly.

### Mine tailing, soil, and pine needle processing and analysis

Mine tailings, reference soils, and pine needles were placed in convection ovens at 60 °C and dried to a constant mass. Next, the tailings and reference soils were sieved to ≤ 2 mm particle size to break up soil aggregates and remove rock fragments and large organic materials such as roots. Mine tailings and reference soils were analyzed for organic matter (%OM) via loss on ignition (LOI) analyses, soil pH, and particle analysis using the same methods described in Sect. “Stream bottom sediments” for bottom sediments. Mine tailings, reference soils, and pine needles were digested for pseudototal concentrations of macronutrients (Ca, Mg, K, P), micronutrients (Cu, Zn), toxic elements (As, Cd, Pb, U), and other pyrite-forming elements (S, Fe) following EPA Method 3050B open vessel hot plate digestion and analyzed with an Agilent 5800 ICP-OES and an Agilent 7900 × ICP-MS. As a brief description of the strong acid digestion process, 5 mL of reverse aqua regia (9 HNO3: 1 HCl) was added to 2.0 ± 0.1 g of soil sample in 50-mL centrifuge tubes. In total, 0.5 ± 0.01 g of pine needles was measured into ceramic crucibles and ashed in a muffle furnace at 550 °C for 8 h, after which they were transferred to 50-mL tubes and digested with 5 mL of reverse aqua regia. The plant and soil samples were placed in a tube rack heater at 80 °C for 1 h. After, the samples were diluted to 50 mL with deionized (DI) water. Three-gram aliquots of the diluted solutions were further diluted with DI water to a 15 g final volume before analysis. A duplicate sample, a blank sample, and a 0.100 ± 0.001 g sample of standard reference material (SRM) from the U.S. National Institute of Standards and Technology were included every twenty one soil sample to evaluate ICP-MS and ICP-OES performances. SRM 1515 or 1547 was the reference material for the plant samples and SRM 2710a for the soil samples.

### Mine tailings, biochar, and lime addition column experiment

A set of columns were conducted to evaluate if biochar and lime could reduce the mobility of PTEs in leachate to stream waters from mine tailings, using methods of periodic leachate collection in re-packed columns similar to Khoeurn et al. (2019), Embile et al. (2018), and following guidelines from ASTM D4874-95 (ASTM 2014). We chose mine tailings from the Jenkins Mine due to the highest concentrations of As, Cu, and Pb measured both in the mine tailings and in their adjacent Contrary Creek stream water. Twelve columns (45-cm height and 6.9-cm diameter) were packed with 1.5 kg of mine tailings from the Jenkins mine site. The soils were secured from the bottom of each column with 20-µm mesh. Columns were wetted from the bottom to fill air voids commonly created from top-down wetting. Every 3 days, a flushing event occurred in which 360 mL of 10 mM NaCl and 10 mM CaCl_2_ solution was added to each column to mimic rainfall events and allow for partially saturated hydrologic conditions that occur for the exposed mine tailings. In total, there were twenty 3-day flushing events over a 60-day period. After the initialization period of 5 flushing events over 15 days, four different treatments were applied to the columns. Three control columns served as controls of leachate and received no biochar or lime but had the top 10 cm churned. The remaining nine columns received either 0.92 tonnes/ha treatment (1.5 g of biochar and lime added to the 1.5 kg of mine tailing), 9.2 tonnes/ha (15 g of biochar and lime added to the 1.5 kg of mine tailing), or 92 tonnes/ha treatment (150 g of biochar and lime added to the 1.5 kg of mine tailing). The biochar was made from mixed softwood and hardwoods produced by SWVA BioChar company (3000 mg/kg Ca, 2000 mg/kg Fe, P 250 mg/kg, PTEs were < 10 mg/kg, CEC measured at 2.9 cmol_c_/kg) and Espoma Lime (CaO 29%, MgO 17%, CaCO_3_ 52%). The biochar and lime were physically mixed into the top 10 cm of mine tailings to simulate mixing application.

Following every flushing event, the leachates collected within 18 h were analyzed for their pH and ORP using a Fisher Accumet AB315 pH probe after the rainwater solution was applied. The total leachates were then weighted for their total mass and filtered using 0.45-µm Whatman membrane filters. Samples were then acidified with 1 mL of 35% HCl and then diluted 1:1 with 2% HNO_3_ for elemental analysis.

### Statistical analysis

Descriptive statistics were calculated using MATLAB. Mean PTE and P concentrations and soil properties are given ± 1 standard error in the text, tables, and figures. Due to limited sample sizes, the Kruskal-Wallis test was used to identify statistically significant differences among three or more groups while the Wilcoxon rank-sum test was used to identify statistically significant differences between two groups. Relationships between PTEs and P concentrations with watershed properties (agricultural land-use, urban development land-use, number of former mines) and physicochemical properties (% SOM, pH, and Fe concentrations) were evaluated using stepwise regressions and principal component analysis in SPSS.

## Results and discussion

### Stream waters

**Fig. 2:**
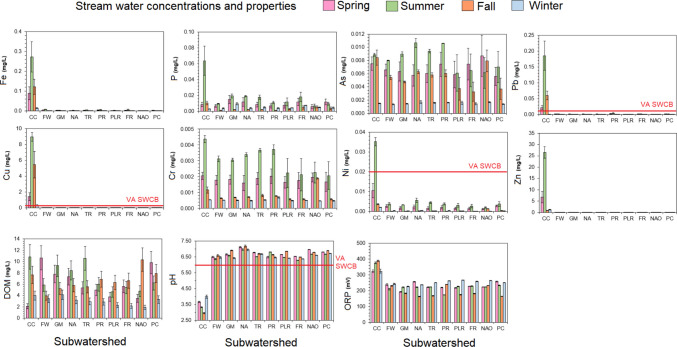
Stream water concentrations across the nine subwatersheds and lake outflow (NAO) from March 2024 to March 2025. Error bars are standard error across the sampling locations within each subwatershed. The red line indicates an exceedance of the Virginia State Water Control Board (SWCB) water quality standards for contaminants in surface waters (9VAC25-260-140) and minimum surface water pH of 6.0 (9VAC25-260-50)

Due to the seasonal differences in precipitation and stream discharge rates observed from USGS data, stream water concentrations were first evaluated on a seasonal basis (Fig. 2). Stream water PTE and nutrient concentrations were typically highest during the summer season and typically lowest during the winter season. Across all streams and seasons, average stream water concentrations were the following: P 0.009 ± 0.001 mg/L, Fe 0.019 ± 0.011 mg/L, Cr 0.002 ± 0.001 mg/L, Ni 0.003 ± 0.001 mg/L, Cu 0.409 ± 0.221 mg/L, Zn 0.009 ± 0.001 mg/L, As 0.009 ± 0.001 mg/L, and Pb 0.009 ± 0.001 mg/L. These average stream water concentrations are below ranges typically observed for surface waters impacted by acid mine drainage (see Johnson and Hallberg 2005; Yuan et al. 2022 in which As, Cr, Cu, Mn, and Zn commonly exceeded 1 mg/L) and eutrophic surface waters from agricultural operations (e.g., Correll 1998 and Evans-White et al. 2013 found that total P commonly exceeded 0.050 mg/L in areas with eutrophication). Stream water Cd and U were also analyzed but were < 0.0002 mg/L and not thus not considered in our analyses.

Virginia Water Quality Standards does not have an overarching P criterion, but we compared concentrations to the specific Lake Anna criterion for total P of 0.03 mg/L in VA code § 9VAC25-260 from 1992. Across the subwatersheds, stream water P concentrations were below the 1992 criterion concentration. Only stream water P concentrations for CC during the summer season exceeded the 1992 water quality criterion of 0.03 mg/L criterion content (Fig. 2). These results highlight that watershed management practices have decreased stream water P concentrations to meet goals necessary for protecting the greater Chesapeake Bay watershed from eutrophication from agricultural areas and domestic sources.

Stream water concentrations of PTEs revealed several elements were elevated above typical freshwater stream concentrations and exceeded Virginia State Water Control Board (SWCB) Water Quality Standards VA Code § 9VAC25-260-140 Criteria for surface water. Stream water concentrations of Fe, Cr, and Zn were not elevated above Virginia SWCB water quality standards (Fig. 2), although CC had elevated Fe and Zn above typical freshwater stream concentrations (Kabata-Pendias and Mukherjee 2007). Wood (1973) measured stream waters Fe concentrations at CC in winter of 1972 and measured 0.50 mg/L, which are higher than our 2024 observations of 0.05 to 0.27 mg/L. Moreover, Dagenhart (1980) and Hinkle (1982) measured ranges of stream water concentrations between 1976 and 1980 following remediation of Cu 0.02 to 7.4 mg/L, Pb 0.01 to 0.47 mg/L, and Zn 0.01 to 18.3 mg/L, which are all comparable to our measured Cu, Pb, and Zn concentrations for CC. These results suggest that AMD increased Fe leaching to surface waters at CC, which are lower since the implementation of additional remediation actions following the studies by Wood (1973), Dagenhart (1980), and Hinkle (1982). Stream water Pb, Cu, and Ni concentrations were below standards for all other subwatersheds except for CC which exceeded Virginia SWCB Water Quality Standards for chronic freshwater criteria for aquatic life VA Code § 9VAC25-260-140 for three seasons (spring, summer, fall) for Cu and Pb and only summer for Ni. Stream water values of Pb, Cu, and Ni were highest during the summer with maximum concentrations in June with Pb at 0.241 ± 0.036 mg/L, Cu at 11.4 ± 0.4 mg/L, and Ni at 0.038.2 ± 0.014 mg/L. Regional Pb, Cu, and Ni values for the other eight subwatersheds were multiple orders of magnitude lower: Pb at 0.0009 ± 0.0001 mg/L, Cu at 0.004 ± 0.001 mg/L, and Ni at 0.0016 ± 0.0002 mg/L. Stream water As concentrations did not exceed Virginia SWCB Water Quality Standards for chronic freshwater criteria of 0.150 mg/L. However, nearly all sites approached or exceeded As concentrations considered hazardous for drinking of 0.010 mg/L by the Virginia SWCB Water Quality Standard VA Code § 9VAC25-260-140 and UN FAO (see Frisbie and Mitchell 2022). Since Lake Anna is not a human drinking water reservoir, this is unlikely to apply or affect human communities living within the watershed. Overall, our stream water results show that metal pollution from historical mining appears to be localized to CC while the other eight subwatersheds are below concentrations known to cause chronic toxicity to freshwater ecosystems or chronic non-potable human exposure.

Stream water pH criteria comes from Virginia SWCB law 9VAC25-260-50, which provides a pH range of 6.0 to 9.0 for Class III (coastal and piedmont zones) waters. We observed that CC fell far below this criterion during all four seasons, with the lowest pH observed in during summer (pH = 2.97 ± 0.05) (Fig. 2). Wood (1973) measured stream waters at CC in winter of 1972 between pH 2.9 and 3.5, which match our 2024 observations. Our stream water Fe results suggest that AMD has diminished but the low stream water pH suggests the legacy effects of AMD still occur. This may be due to greater Fe complexation by organic matter (e.g., Suteerapataranon et al. 2006) or secondary oxyhydroxide precipitation (e.g.,Jönsson et al. 2006; Ying et al. 2022) without neutralization of the acidity. Decreases in metals without significant increases in pH agree with Boult (1994) in which metals can behave conservatively with pH indicating other factors can drive both solubility of metals from AMD and legacy effects promoting acidification of stream waters. Stream water pH did not exhibit a consistent seasonal difference among the nine subwatersheds. Stream water ORP was significantly higher for CC (353 ± 17 mV) than the other subwatersheds ranging (223 ± 30 mV). The higher stream water ORP was expected due to the inverse relationship with pH and ORP (Stumm and Morgan 2013) but opposite of findings from more intensive forms of AMD in which oxidation of reduced Fe from mines can decrease stream water ORP.

**Fig. 3:**
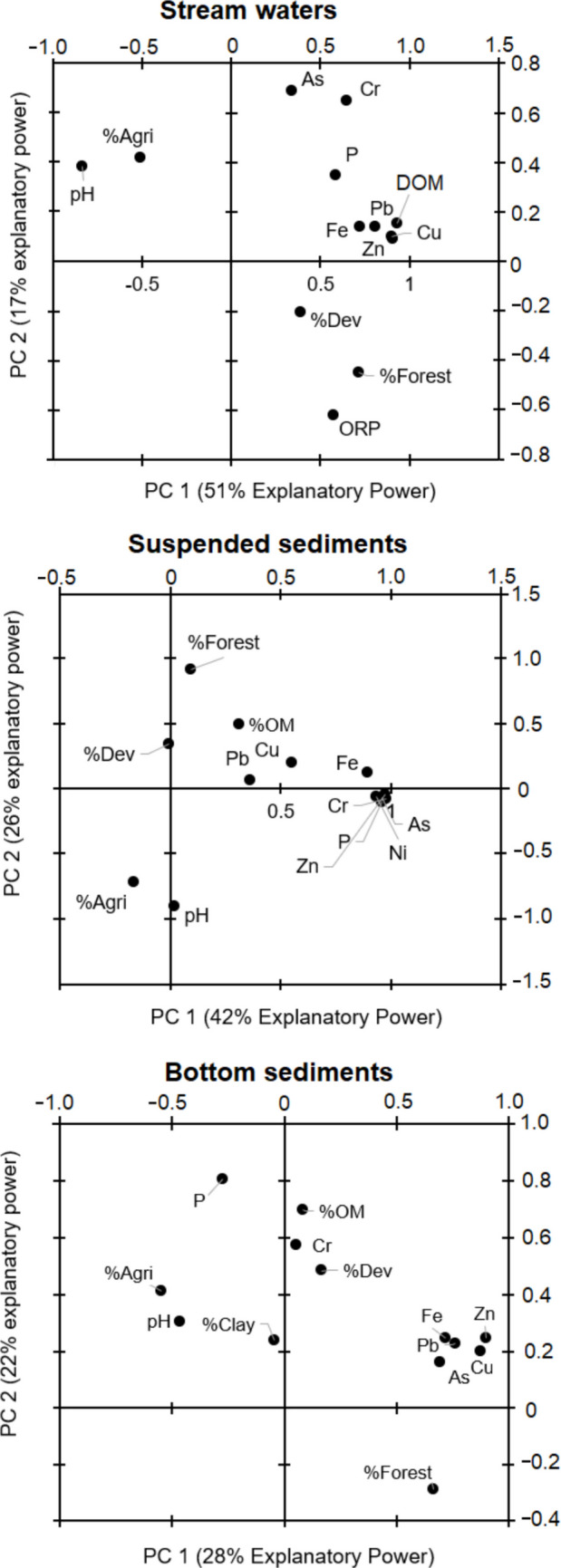
Principal component analysis of potentially toxic element and P concentrations, chemical properties (pH and %OM or DOM) and watershed land cover data for agriculture (%Agri), developed (%Dev), and forested (%Forest) with sampling time or location used as replicates for each sampling site

To evaluate the relationship between land-use and physicochemical properties with stream water dissolved elemental concentrations, we conducted principal component analysis for dimensional reduction and illustrate their relative associations (Fig. 3). PC1 had 51% explanatory power and grouped most of the stream water PTEs and P concentrations with stream water DOM, ORP, and watershed land use of %developed and %forest cover. Stream water pH and %agricultural land use were negatively associated with PTEs and P concentrations. Considering PC2 which only had 17% explanatory power, stream water pH, P, and %agricultural land use were associated while ORP, %developed, and %forest were negatively associated. From the PCA (Fig. 3), we can see that most PTEs and DOM were strongly associated together, while land-uses and pH-ORP were negatively associated with each other. These relationships suggest DOM enhanced PTE transport due to chelation or co-occurred during flushing events while lower pH was negatively associated with oxygen within the water column. Stream water P was only weakly associated with watershed %agricultural land-use cover. The P concentration in watersheds was not associated with %agriculture as originally hypothesized, though its association with %developed suggests that urban area and areas with cut trees have a greater influence on the transport of dissolved P in streams compared to the proportion of farmland. Stream water As and Cr were only weakly associated with pH and DOM.

### Suspended sediments

**Fig. 4:**
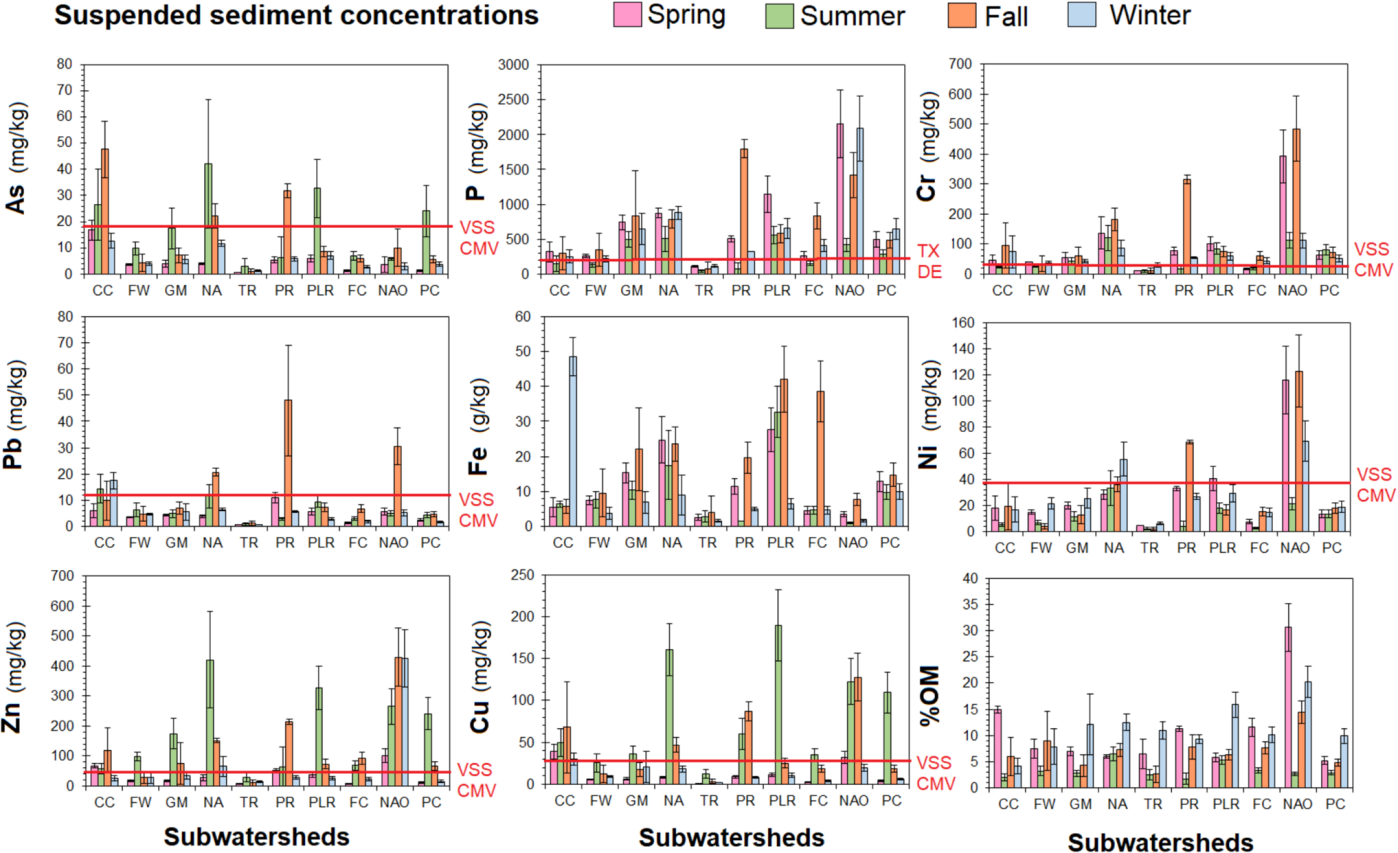
Suspended sediment concentrations, mass of elements collected on 0.45-μm filters per liter, across the nine subwatersheds and lake outflow (NAO) across seasons of 2024. *N*= 3 for each sampling location within subwatershed. The red line indicates an exceedance of the Virginia Department of Environmental Quality (DEQ) for Virginia soils and sediments (VSS) contaminated media variance (CMV) ecological screening value

Suspended sediments > 0.45 µm were filtered from stream water to examine particulate driven transport of P and PTEs across the nine subwatersheds (Fig. 4). Across all streams and seasons, average suspended sediment concentrations were the following: P 583 ± 172 mg/kg, Fe 12,360 ± 3930 mg/kg, Cr 86 ± 33 mg/kg, Cu 37 ± 15 mg/kg, Ni 25 ± 9 mg/kg, Zn 101 ± 40 mg/kg, As 139 ± 59 mg/kg, and Pb 7 ± 3 mg/kg. Our suspended sediment concentrations were far below concentrations reported in other Zn–S mining zones, such as Clear Creek in the Colorado Front range which had far higher suspended sediment concentrations of Cu (1000–3000 mg/kg), Zn (7000–11,000 mg/kg), and Fe (53–192 g/kg) as reported by Butler et al. (2008). As of 2025, the Commonwealth of Virginia does not have regulations for elements within suspended sediments; thus, we leverage the Virginia Department of Environmental Quality (DEQ) for Virginia soils and sediments (VSS) contaminated media variance (CMV) ecological screening value. Suspended sediment concentrations of As, Cr, Pb, Ni, Zn, and Cu exceeded CMV values for ecological screening for several of the subwatersheds, most consistently at CC, NA, PR, PLR, and NAO (Fig. 4). Suspended sediment Cd and U were analyzed but were < 0.5 mg/kg and not thus not considered in our analyses. Unlike stream water concentrations, there were limited seasonal differences. Suspended sediment As, Cu, and Zn concentrations were generally higher during summer while Cr and Pb were higher during the fall, which is likely due to lower flow rates punctuated with large storm events. %OM concentration of the suspended sediment was higher during the winter likely due to greater precipitation, higher discharge rates, and more subsurface flow.

The US EPA and Virginia DEQ do not have a bottom sediment P concentration criteria at the time of this experiment. Instead, we have followed the definition of excessive P concentrations of 150 mg/kg total P for sediments made by Texas A&M AgriLife Extension Service (see Provin & Pritt 2008) and Delaware state law 3 Del. C. § 2247 as a proximal regulatory limit (Nutrient management certification regulations n.d.). Suspended sediment P concentrations in all subwatersheds except Terry’s Run (TR) exceeded the 150 mg/kg P concentration.

It is critical to note that high PTE and P concentrations within suspended sediments may not constitute a large flux if sediment mass per liter of stream water is low to negligible. To investigate the relative importance of stream water and suspended particles within the stream water, we scaled the mass of elements per liter of stream water using the mass of suspended sediments per liter in Table 2. In this comparison, we found that suspended sediments carried significantly higher mass of P, Fe, As, Cu, and Zn per liter across all streams by nearly two orders of magnitude when compared to dissolved sediments (Table 2). Similarly, suspended sediments carried significantly higher mass of Pb and Ni per liter across all streams except for CC. At Contrary Creek (CC), Pb and Ni mass was not significantly different between stream water and suspended sediments and Cu mass was significantly greater in stream water by over an order of magnitude (Table 2). Surprisingly, dissolved organic matter and suspended sediment organic matter was only significantly different for one subwatershed, PC. Our results show that suspended sediments are generally the most dominant transport for PTEs and P across the subwatersheds except for CC, which agrees with the AMD literature that under pH increases solubility of metals promoting dissolved transport (e.g.,Equeenuddin et al. 2013; Ying et al. 2022). At moderate to weakly acidic pH, sorption to Al and Fe oxyhydroxides and organic matter drives the transport of metals from mine tailings and soils to streams (e.g.,Equeenuddin et al. 2013; Ying et al. 2022).

**Table 2:** Comparison of mean annual dissolve streamwater concentrations and suspended sediment concentrations for select PTE

Subwatershed	Phosphorus		Arsenic		Lead		Copper	
	Stream water	Suspended sediment	Stream water	Suspended sediment	Stream water	Suspended sediment	Stream water	Suspended sediment
	mg/L	mg/L	mg/L	mg/L	mg/L	mg/L	mg/L	mg/L
CC	0.02±0.01	0.95±0.17*	0.01±0.01	0.35±0.16*	0.07±0.04	0.05±0.01	4.1±1.9*	0.20±0.06
FW	0.01±0.01	1.87±0.88*	0.01±0.01	0.47±0.19*	0.01±0.01	0.03±0.01*	0.00±0.00	0.10±0.03*
GM	0.01±0.01	2.48±0.67*	0.01±0.01	0.40±0.19*	0.01±0.01	0.02±0.01	0.00±0.00	0.07±0.02*
NA	0.01±0.01	1.90±0.35*	0.01±0.01	0.84±0.50*	0.01±0.01	0.03±0.02	0.00±0.00	0.18±0.11*
TR	0.01±0.01	1.45±0.23*	0.01±0.01	0.41±0.24*	0.01±0.01	0.02±0.01	0.00±0.00	0.09±0.05*
PR	0.01±0.01	1.19±0.45*	0.01±0.01	0.27±0.08*	0.01±0.01	0.03±0.01*	0.00±0.00	0.12±0.07*
PLR	0.01±0.01	1.80±0.54*	0.01±0.01	0.37±0.15*	0.01±0.01	0.02±0.01	0.00±0.00	0.12±0.06*
FR	0.01±0.01	2.21±0.72*	0.01±0.01	0.26±0.11*	0.01±0.01	0.02±0.01	0.00±0.00	0.10±0.06*
PC	0.01±0.01	2.28±0.34*	0.01±0.01	0.77±0.41*	0.01±0.01	0.02±0.01	0.00±0.00	0.17±0.08*
NAO	0.01±0.01	1.69±0.35*	0.01±0.01	0.47±0.33*	0.01±0.01	0.01±0.01	0.00±0.00	0.12±0.08*
	Iron		Nickel		Zinc		Organic matter	
	Stream water	Suspended sediment	Stream water	Suspended sediment	Stream water	Suspended sediment	Stream water	Suspended sediment
	mg/L	mg/L	mg/L	mg/L	mg/L	mg/L	mg/L	mg/L
CC	0.18±0.10	41±20	0.013±0.007	0.05±0.01	8.66±3.13	0.29±0.10	6±2	7±3
FW	0.004±0.001	54±23	0.002±0.000	0.07±0.02	0.007±0.002	0.29±0.11	6±2	7±1
GM	0.002±0.000	52±21	0.002±0.000	0.06±0.01	0.002±0.000	0.28±0.13	7±1	7±2
NA	0.001±0.000	50±14	0.002±0.000	0.10±0.02	0.002±0.000	0.50±0.28	6±1	8±2
TR	0.003±0.001	52±16	0.002±0.000	0.06±0.01	0.004±0.001	0.27±0.08	6±2	6±2
PR	0.003±0.000	59±23	0.002±0.000	0.06±0.02	0.004±0.001	0.20±0.06	5±1	7±2
PLR	0.001±0.000	86±25	0.001±0.000	0.06±0.01	0.002±0.000	0.26±0.12	4±1	8±3
FR	0.003±0.000	71±23	0.001±0.000	0.05±0.01	0.003±0.000	0.28±0.13	5±1	8±2
PC	0.001±0.000	6±2	0.001±0.000	0.13±0.03	0.002±0.000	0.55±0.15	5±2	17±6
NAO	0.002±0.000	44±12	0.002±0.000	0.06±0.01	0.002±0.000	0.30±0.18	7±1	6±1

We conducted principal component analysis to evaluate suspended sediment PTE and P concentrations with physicochemical and watershed properties (Fig. 3). PC1 had 42% explanatory power and grouped most of the stream water PTEs and P concentrations closely together, except Pb and Cu to a lesser degree. Suspended sediment Pb and Cu were more closely related to DOM. Watershed %agriculture was negatively associated with higher suspended sediments. PC2 had 26% explanatory power and was largely driven by watershed land-use in which %forest cover and %developed were negatively associated with %agriculture and pH. From the PCA, we can see that suspended sediment PTEs and DOM were strongly associated together, while land-uses and pH were negatively associated with each other. Similar to stream water, DOM likely increased PTE due to chelation or co-occurred during flushing events with greater sediment. The greater the forest content, suspended sediment had lower pH likely from organic acids from the trees.

### Bottom sediments

**Fig. 5:**
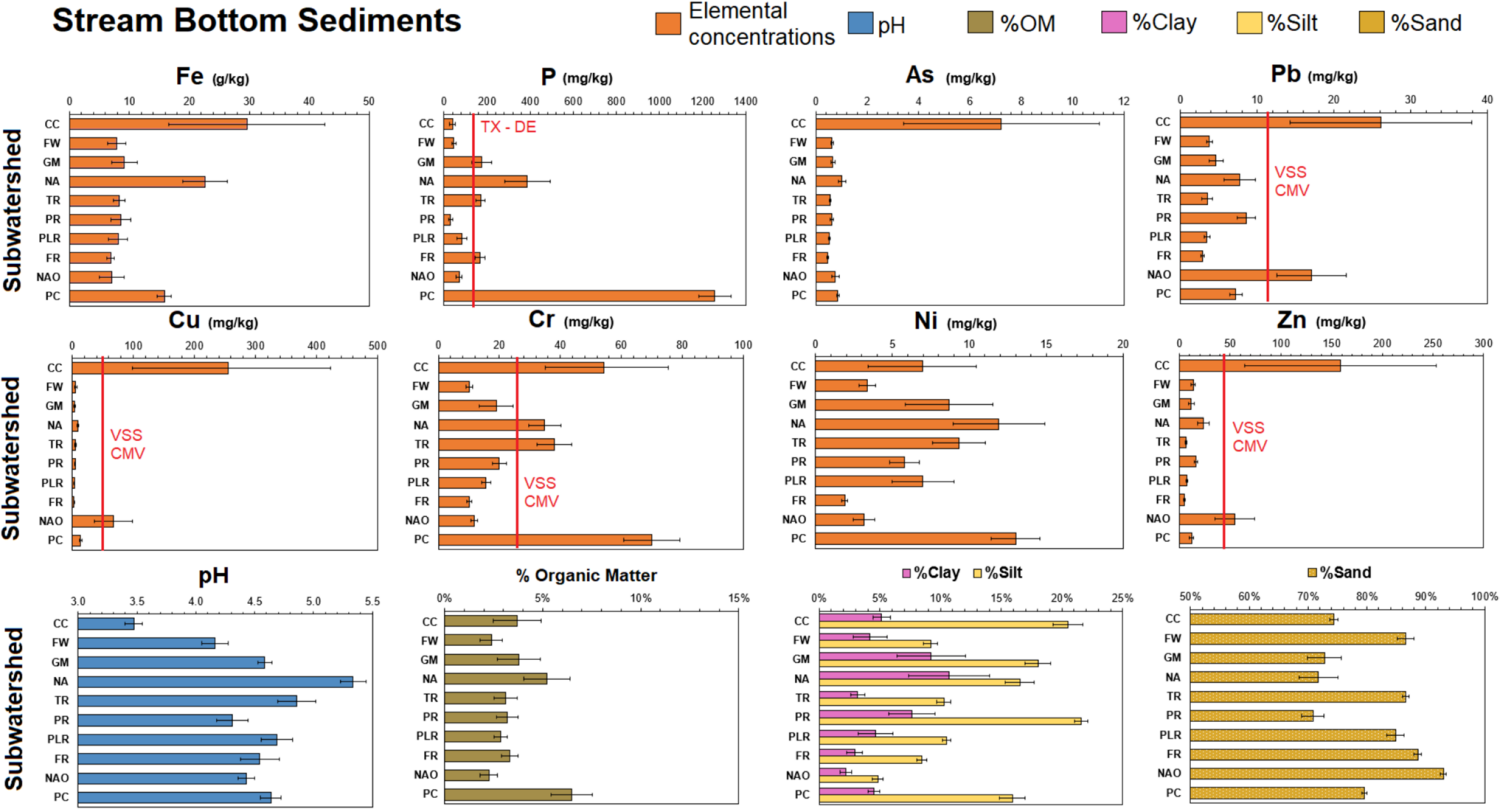
Bottom sediments PTE concentrations and physicochemical properties from across the nine subwatersheds and lake outflow (NAO). Bottom sediments were collected stream eddys. *N*= 9 for each sampling location within each subwatershed. The red line indicates an exceedance of the Virginia Department of Environmental Quality (DEQ) for Virginia soils and sediments (VSS) contaminated media variance (CMV) ecological screening value

Bottom sediments were collected from eddy pools at each of the streams at three to nine different locations with each subwatershed to assess potentially mobile pools of P and PTEs (Fig. 5). Across all subwatersheds, average bottom sediment concentrations were the following: P 242 ± 34 mg/kg, Fe 12,380 ± 2860 mg/kg, Cr 28 ± 6 mg/kg, Cu 37 ± 14 mg/kg, Ni 7.1 ± 1.7 mg/kg, Zn 31 ± 13 mg/kg, As 1.3 ± 0.4 mg/kg, and Pb 28 ± 6 mg/kg. Bottom sediment Cd and U were analyzed but were < 0.5 mg/kg and not thus not considered in our analyses. Bottom sediment concentrations of Pb, Cu, Zn, and Fe were within the range of lake sediments from Lake Anna (Odhiambo et al. 2013), and Zn, Cu, Ni, and Pb concentrations were similar to Chickahominy River sediments in eastern Virginia (Hupp et al. 1993). Lastly, bottom sediment concentrations of Cr, Cu, Ni, Pb, and Zn were comparable to sediments measured in the Elizabeth River in southern VA (Conrad et al. 2007). The bottom sediment PTE concentrations also are generally comparable to concentrations to streams impacted by mining in Korean, Brazil, Nigeria, and India with their bottom sediment concentrations ranging as follows: Cr 11 to 386 mg/kg, Cu 6 to 137 mg/kg, Ni 0.5 to 124 mg/kg, Zn 9 to 195 mg/kg, and Pb 3 to 38 mg/kg (Equeenuddin et al. 2013).

We compared bottom sediment PTE concentrations with Virginia Department of Environmental Quality (DEQ) for Virginia soils and sediments (VSS) contaminated media variance (CMV) ecological screening value to evaluate potential hazard to aquatic organisms. Bottom sediment Pb, Cu, and Zn concentrations at CC and NAO exceeded concentrations by the VSS CMV screening values to be potentially hazardous ecologically (Fig. 5). No other streams exceeded the VSS CMV screening values of Pb, Cu, and Zn concentrations (Fig. 5). These results highlight that sediment not actively being transported but temporarily stored within the stream bottom contain PTEs that can negatively impact aquatic invertebrates or plants. Unfortunately, quantifying impacts on aquatic ecosystems was beyond the scope of this project, but reports from Virginia State Water Control Board in 1982 indicated that reclamation efforts have decreased toxic metal concentrations and loads but biological recovery within the stream had been negligible (Hinkle 1982).

We have followed the Texas A&M AgriLife Extension Service (Provin & Pritt 2008) Delaware state law 3 Del. C. § 2247 as a proximal regulatory limit to evaluate excessive P concentrations of 150 mg/kg total P for sediments (Nutrient management certification regulations n.d.). Using this criterion, it was found that total P concentrations for five subwatersheds had excessive amounts of P in bottom sediments (GM, NA, TR, FR, PC) (Fig. 4). In particular, bottom sediments in the North Anna River (NA) and Pamunkey Creek (PC) subwatersheds were found to have total P concentrations 606 ± 81 mg/kg and 1257 ± 13 mg/kg, respectively (Fig. 5). Although Pamunkey Creek had the highest bottom sediment P concentrations, stream water P in Pamunkey Creek waters never exceeded the criteria. Surprisingly, bottom sediment P concentrations did not correspond with suspended P concentrations. In particular, bottom sediment P concentrations were greatest at PC but suspended sediment P concentrations at PC were average compared to the other subwatersheds, which indicates a disconnect between more organic sediments stored in the bottom of the streams compared to more siliceous being actively transported.

We evaluated bottom sediment physicochemical properties to examine if particle size, organic matter content, or pH would affect sorption. Bottom sediment %OM did not vary widely, ranging from 2.9 to 6.5%. The higher %OM content for PC and NA subwatersheds corresponded with higher P concentrations. Clay content ranged from 2.2 to 10.7%, which was highest at GM and NA subwatersheds, which also corresponded with higher bottom sediment P concentrations. Across subwatersheds, bottom sediment pH at most sites had relatively acidic sediments, ranging from a pH of 3.4 to 5.6 (Fig. 5) and bottom sediment pH was lowest at CC site with 3.5 pH. When compared to the EPA minimum criteria for freshwater sediments for plant growth of pH = 5.5, nearly all subwatersheds were below this threshold, in particular CC. The low pH for bottom sediments across Lake Anna highlights several aspects: (1) the Lake Anna watershed has lithology that generates acidic surface waters; (2) the sediments have been intensively weathered and offer few weatherable minerals to generate sufficient buffering capacity; (3) human agricultural activities are enhancing organic matter and organic acids that further acidify the subwatersheds. Mineralogical analyses of the stream bottom sediments were not conducted nor were total digestions applied to quantify the extent of chemical weathering through weathering indices; thus, we cannot conclude on the extent of loss of base cation bearing minerals from bottom sediments across the watershed.

We conducted PCA (Fig. 3) to evaluate bottom sediment PTE and P concentrations with physicochemical and watershed properties. PC1 had 28% explanatory power and bottom sediment Fe, Pb, As, Cu, and Zn concentrations, and %forest were closely together, while Cr, %OM, and %developed were grouped together. From the PC1, we can see that bottom sediment PTEs were strongly associated together along with the amount of forest not agriculture or development across watersheds. Also, from PC1, we observe that watershed %agriculture, pH, %OM, and bottom sediment P concentrations were more closely related. This agrees with our hypothesis that agricultural land-use in a watershed controls sediment P. Surprisingly, the clay content of the bottom sediment was not strongly associated with bottom sediment PTE or P concentrations. PC2 had 22% explanatory power and was largely driven by watershed land-use %forest cover. Similar to stream water PTE concentrations, bottom sediment PTE concentrations appear to be influenced in some way by %forest and %developed. This indicates a possible connection between forest cover and PTE abundance that was not originally hypothesized.

### Dissolved and sediment-bound PTE and P watershed export

**Fig. 6:**
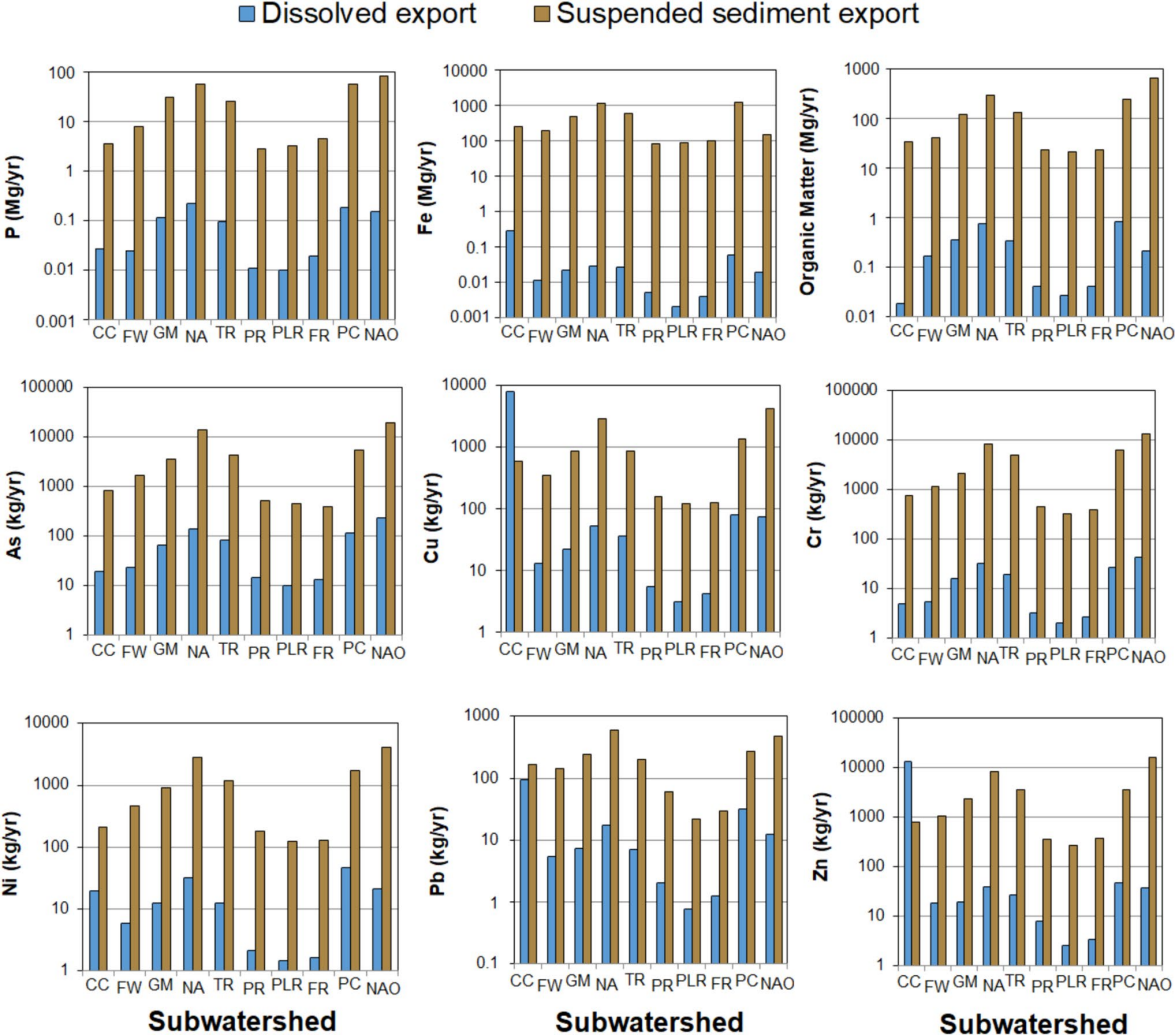
Total annual dissolved and suspended sediment PTE and P masses from the nine subwatersheds and from the Lake Anna reservoir outflow (NAO). Total annual export rates from March 2024 to March 2025 were determined using measured stream water and suspended concentrations and USGS-monitored discharge rates

Total annual dissolved and suspended sediment-bound PTE and P were determined using monthly discharge rates from the USGS stream gauges and field samples to compare the relative importance for transport mechanism and among the subwatersheds (Fig. 6). Total dissolved and suspended sediment export of DOM, Fe, P, and PTEs (As, Cr, Cu, Pb, Ni, and Zn) was consistently greatest for the largest subwatersheds, North Anna River (NA), Pamunkey Creek (PC), and the outflow from the Lake Anna Reservoir (NAO) and generally lowest at the smallest subwatersheds Pleasant Run (PLR), Foremost Run (FR), and Pigeon Run (PR) (Fig. 6). This simply shows that larger subwatersheds with more soils, regolith, and human activities have more water and sediment being transported in their streams. However, there were some exceptions that did not follow the watershed size-controlled trend. Dissolved export of Cu and Zn for CC was greater than the larger subwatersheds by nearly two orders of magnitude and greater than suspended sediment transport across the other subwatersheds and Lake Anna outflow. Dissolved export of Pb was also highest for CC subwatershed and was similar or greater than to suspended sediment bound Pb export for several of the other watersheds. This highlights the substantial impact from exposed mine tailing at CC, described in greater detail in Sect. “Mine tailing pine needle PTE and nutrient concentrations.” Despite the presence of As in sulfides found at CC (see Sect. “Mine tailing pine needle PTE and nutrient concentrations”), As was not elevated in dissolved or suspended sediment for CC compared to the other subwatersheds. Lastly, we examined the retention of PTEs and P within Lake Anna by comparing the summed total annual export into Lake Anna by the nine subwatersheds and the total annual export from Lake Anna from the outflow. Our results show that Lake Anna is a net accumulator of dissolved and suspended Fe, P, and most PTEs with 48% up to 99% retention rates, with a few exceptions. Only 37%, 43%, and 21% of suspended sediment As, Cu, and Zn, respectively, were retained within Lake Anna reservoir. This result highlights that Lake Anna retains most of the dissolved and suspended sediments, most likely through burial in lake bottom sediments (see Odhiambo et al. 2013; Butler et al. 2023). By comparing the subwatershed export and the Lake Anna outflow export, Lake Anna is a sink for dissolved (79% retention) and suspended sediment P (56% retention) but still releasing over 0.55 Mg/year dissolved P and 107 Mg/year suspended sediment P to the North Anna River, York River watershed, and potentially to the Chesapeake Bay.

To understand differences in land-use and soils, the dissolved and suspended sediment export rates were normalized to their respective watershed area for comparisons across small and large watersheds (Table 3). For dissolved export rates, the largest watersheds no longer had the highest area-normalized dissolved or suspended sediment export for most PTEs and P (Table 3). Instead, CC had the highest export rate for Fe and most PTEs (Cu, Pb, Ni, and Zn) while Goldmine creek (GM), Freshwater Creek (FW), and Foremost Run (FR) had the highest area-normalized dissolved export rates for DOM and P. For area-normalized suspended sediment exports, Goldmine creek (GM), Freshwater Creek (FW), and North Anna River (NA) had the highest area-normalized dissolved export rates for P and several PTEs (As, Cr, Ni, and Zn). Suspended sediment Fe, Cu, and Pb were still greater at CC, likely due to exposed mine tailings with the watershed forming organomineral and Fe oxyhydroxides colloids (e.g.,Jönsson et al. 2006; Ying et al. 2022). The significantly lower area-normalized annual dissolved and suspended sediment export rates for P and PTEs for the Lake Anna outflow highlight net retention within the reservoir.

**Table 3:** Comparison of area-normalized total annual dissolved and suspended sediment export of PTE and P masses on area-normalized annual basis (kg yr^−1^ km^−2^or Mg yr^−1^ km^−2^). Export rates were determined using measured stream water and suspended concentrations and USGS monitored discharge rates

	Area-normalized annual dissolved stream water export								
**Subwatershed**	**DOM**	**P**	**Fe**	**As**	**Cr**	**Cu**	**Pb**	**Ni**	**Zn**
	kg yr^−1^ km^−2^	kg yr^−1^ km^−2^	Mg yr^−1^ km^−2^	kg yr^−1^ km^−2^	kg yr^−1^ km^−2^	kg yr^−1^ km^−2^	kg yr^−1^ km^−2^	kg yr^−1^ km^−2^	kg yr^−1^ km^−2^
CC	1.03	1.5	0.164	1.1	0.28	437	5.31	1.11	764
FW	7.18	1.0	0.005	1.0	0.24	0.57	0.23	0.26	0.8
GM	6.31	2.0	0.004	1.2	0.28	0.39	0.13	0.22	0.3
NA	5.64	1.6	0.002	1.1	0.25	0.39	0.13	0.24	0.3
TR	4.88	1.4	0.004	1.2	0.28	0.51	0.10	0.18	0.4
PR	3.82	1.0	0.005	1.4	0.31	0.50	0.19	0.20	0.7
PLR	3.38	1.2	0.003	1.3	0.26	0.38	0.09	0.18	0.3
FR	4.12	2.0	0.004	1.3	0.27	0.42	0.12	0.16	0.4
PC	6.27	1.4	0.005	0.9	0.20	0.59	0.24	0.34	0.4
NAO	0.24	0.2	0.000	0.3	0.05	0.08	0.01	0.02	0.0
	Area-normalized annual suspended sediment export								
**Subwatershed**	**OM**	**P**	**Fe**	**As**	**Cr**	**Cu**	**Pb**	**Ni**	**Zn**
	kg yr^−1^ km^−2^	kg yr^−1^ km^−2^	Mg yr^−1^ km^−2^	kg yr^−1^ km^−2^	kg yr^−1^ km^−2^	kg yr^−1^ km^−2^	kg yr^−1^ km^−2^	kg yr^−1^ km^−2^	kg yr^−1^ km^−2^
CC	1874	201	14	48	44	33	9.1	12	45
FW	1797	341	9	74	51	15	6.2	20	46
GM	2148	530	9	63	38	15	4.2	16	41
NA	2280	417	9	108	63	22	4.5	21	64
TR	1929	371	9	64	71	12	2.9	17	52
PR	2210	252	8	48	42	15	5.5	17	33
PLR	2610	403	11	58	41	15	2.7	16	34
FR	2370	452	10	40	40	12	2.9	13	38
PC	1822	426	9	41	47	10	2.1	13	27
NAO	769	94	0.2	23	15	5	0.5	5	19

### Mine tailings

**Fig. 7:**
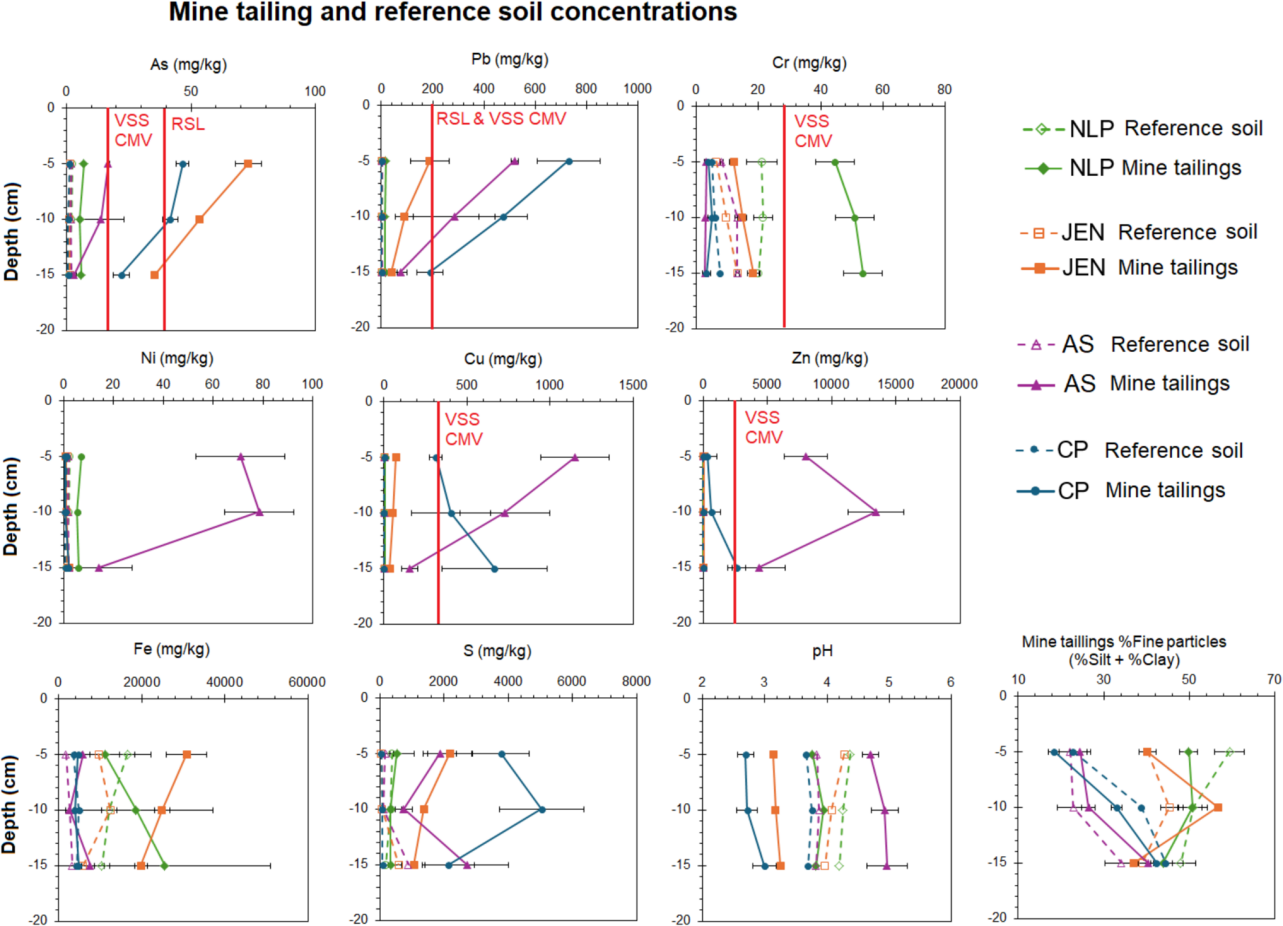
Mine tailing and adjacent reference soil concentrations and physicochemical properties at each of the four former mines studied. *N* = 3 at each mine and adjacent reference site. The red line indicates an exceedance of the 2012 Virginia DEQ State contaminated media variance (CMV) ecological screening value or the US EPA Regional Screening Level

Mine tailings and reference soils (nearby < 20 m of the mine tailing pile) were collected at four former mines (New Luce Prospect, NLP; Jenkins Mine, JEN; Arminus shaft, AS; and Cofer Prospect, CP) within the CC subwatershed. Overall, mine tailings exhibited elevated PTE concentrations, compared with their respective reference site and other reference sites (Fig. 7). Arsenic concentrations for the JEN and CP mine tailings exceeded the US EPA RSL and Virginia CMV values. Arsenic concentrations for the AS mine tailings exceeded Virginia CMV values. Similarly, JEN, CP, and AS mine tailing Pb concentrations exceeded US EPA RSL and Virginia CMV levels. Only NLP mine tailing Cr concentrations exceeded Virginia CMV levels. Mine tailing Cu and Zn concentrations exceeded Virginia CMV levels at AS and CP mines (Fig. 7). Despite the elevated Ni concentration at AS mine tailings, it did not exceed regulatory levels. Surprisingly, mine tailing Fe concentrations were not consistently elevated above their respective reference soil concentrations. Mine tailing S concentrations were significantly higher for CP, AS, and JEN mines than their respective reference soil concentrations, but this was not the case for the NLP mine which had relatively average S concentrations. Our results highlight that the PTE concentrations in the mine tailings in CC are far lower than the original ore (Wood 1973; Dagenhart 1980; Hinkle 1982) and other pyrite mine tailings (see Lindsay et al. 2015). Despite the PTE concentration elevated above reference soils and Virginia contaminated media soil and sediment guidelines, the reclamation efforts and prolonged weathering have decreased PTE concentrations exposed at the surface of the piles. However, the PTE concentrations exceeded contaminated soil and sediment and pose an ecological risk, both to the terrestrial ecosystems that are visibly impacted by sparse vegetation and to the aquatic ecosystems in the Contrary Creek subwatershed.

Mine tailing pH was significantly lower for JEN and CP mine than their reference soil but surprisingly AS mine tailings had significantly higher pH than all reference soils (Fig. 7). Our measured mine tailing pH values match observations made by Wood (1973) of 2.4 to 4.4, showing that AMD has not increased acidity nor has remediation continued to improve exposed mine tailing conditions at the other three mine tailing piles. However, mine tailing organic matter has increased from the measured average of 1.2% ± 0.3% by Wood (1973). Interestingly, mine tailing particle size distribution for the < 2 mm fraction was not significantly different among mine tailing and reference soils. The pH data show that reclamation efforts have been successful at AS, somewhat successful at NLP, but unsuccessful at JEN and CP as mine tailings are still too acidic to effectively support terrestrial life and may still serve as an on-going ecological hazard to the aquatic ecosystems in the Contrary Creek subwatershed. Additional remediation is required and future studies evaluating subsurface flow paths from NLP, JEN, and CP to evaluate their relative contribution of acidity and PTEs to Contrary Creek (CC) is warranted.

### Mine tailing pine needle PTE and nutrient concentrations

**Fig. 8:**
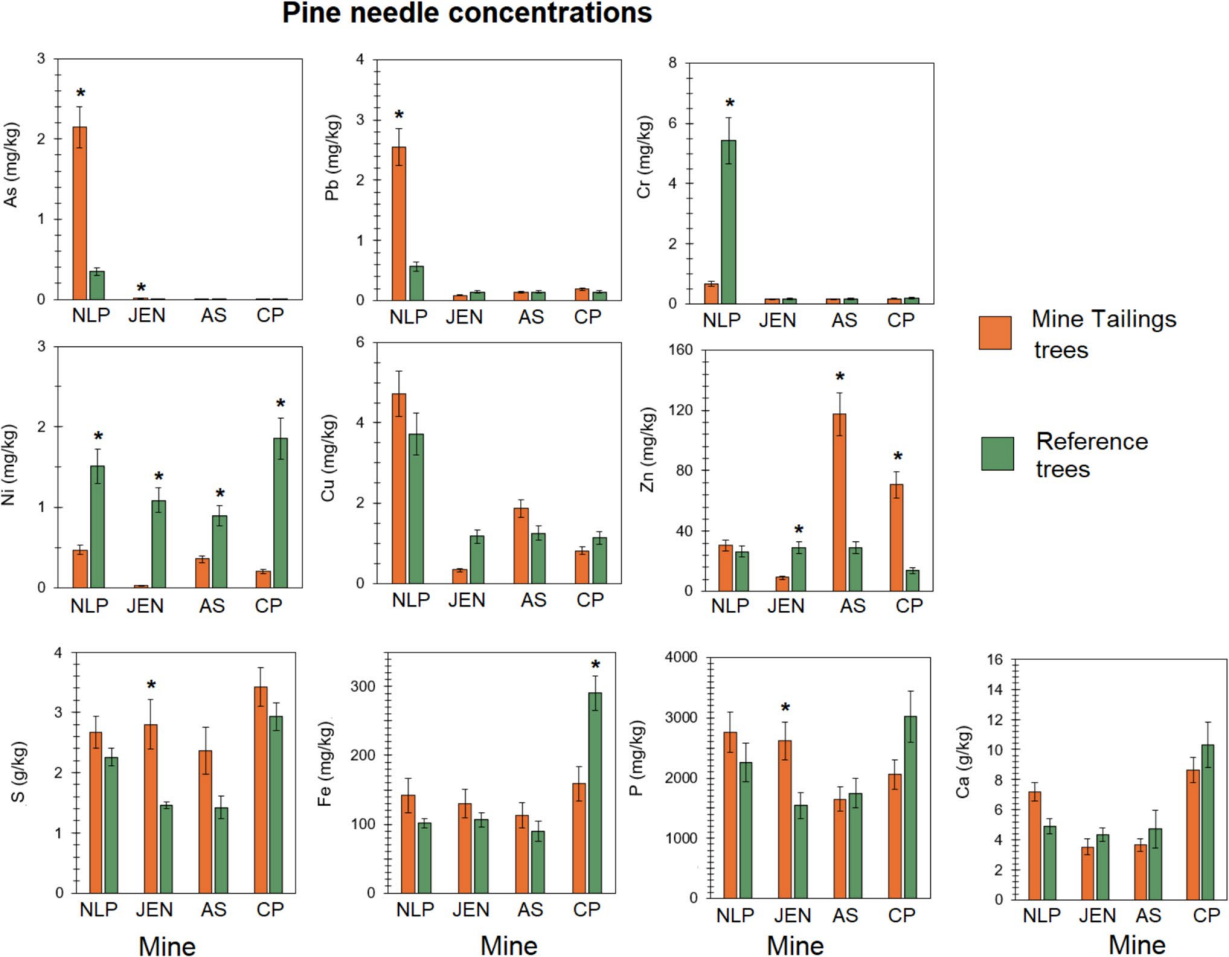
Pine needles concentrations at each of the four former mines studied. Only new growth were sampled. *N* = 10 for each mine and adjacent reference site. Pines at NLP were Virginia pine (*Pinus virginiana*) while JEN, COO, and CP were Loblolly pine (Pinus taeda)

At each of the four mine tailing piles studies, we collected pine needles (from loblolly pine (*Pinus* *taeda*) or Virginia pine (*Pinus virginiana*) from 10 pine trees on the mine tailing piles or periphery and compared them to ten pine trees growing nearby on reference soils to evaluate the impact on their PTE and nutrient accumulation (Fig. 8). We expected higher accumulation of PTEs and lower nutrients for trees on the mine tailings, but we found a more complex, mine-specific and element-specific pattern. Only NLP had significantly higher pine needle As and Pb concentrations for trees on the mine tailings (Fig. 8). Surprisingly, pine needle Ni concentrations were significantly higher for reference soils at all four mines. Pine needle Cr and Cu concentrations were generally not different between mine tailing and reference trees except higher pine needle Cr concentrations at NLP reference soils and higher pine needle Cu concentrations at JEN reference soils (Fig. 8). Pine needle Zn concentrations were mixed, with significantly higher Zn concentrations for mine tailing trees for AS and CP and significantly higher Zn concentrations for reference soils for JEN. Pine needle S concentrations were higher for mine tailing trees but only significantly at JEN. Pine needle Fe concentrations were not significantly different except for reference trees at CP. We expected lower nutrient concentrations in pine needles at mine tailing, but pine needle P and Ca concentrations were not significantly greater than reference soil trees which can likely be attributed to successful reclamation efforts of lime, sludge, and fertilizer application; JEN mine tailing pine trees had higher P than reference soils, and NLP mine tailing pine trees had higher Ca than reference soils (Fig. 8).

Comparing pine needle concentrations measured in this study with values in literature, we find that concentrations are elevated but not beyond previously measured values in other contaminated sites. Pine needle As concentrations were within the range of Korean red pine (*Pinus* *densiflora*) needle concentrations on abandoned minerals processing plant and tailing dump of 1.5 to 7.3 mg/kg (Shin et al. 2019). Pine needle Ca, Cu, P, and Zn concentrations were generally comparable with loblolly pine needles across the southern US, except at AS and CP where Zn concentrations were much greater than data from 110 studies (see Albaugh et al. 2010). Our measured pine needle As, Ca, Cr, Cu, Fe, Pb, and Zn concentrations were similar or lower than black pine needle concentrations in Austria (Zeiner and Juranović Cindrić 2021) but pine needle Ni concentrations in our study were elevated compared with the black pine needles in Austria. Our pine needle Cr, Cu, Ni, and Zn concentrations were similar or lower than Scots pine (*Pinus sylvestris*) and pitch pine (*Pinus rigida*) in lightly polluted areas of Slovakia (Jonczak et al. 2021). This finding was unexpected as acidity should deplete accessible nutrient pools or outcompete their uptake by PTEs (Acharya and Kharel 2020; Wang et al. 2021). This demonstrates a combination of limited bioavailability from the mine tailings and abundant nutrients from previous reclamation applications of fertilizers, liming, and sludge. However, the absolute concentrations can be misleading due to the potential loss of other structural components such as low C or N in biomass leading to an enrichment effect on metal concentrations when scaled to dry biomass.

### Mine tailing column experiment

**Fig. 9:**
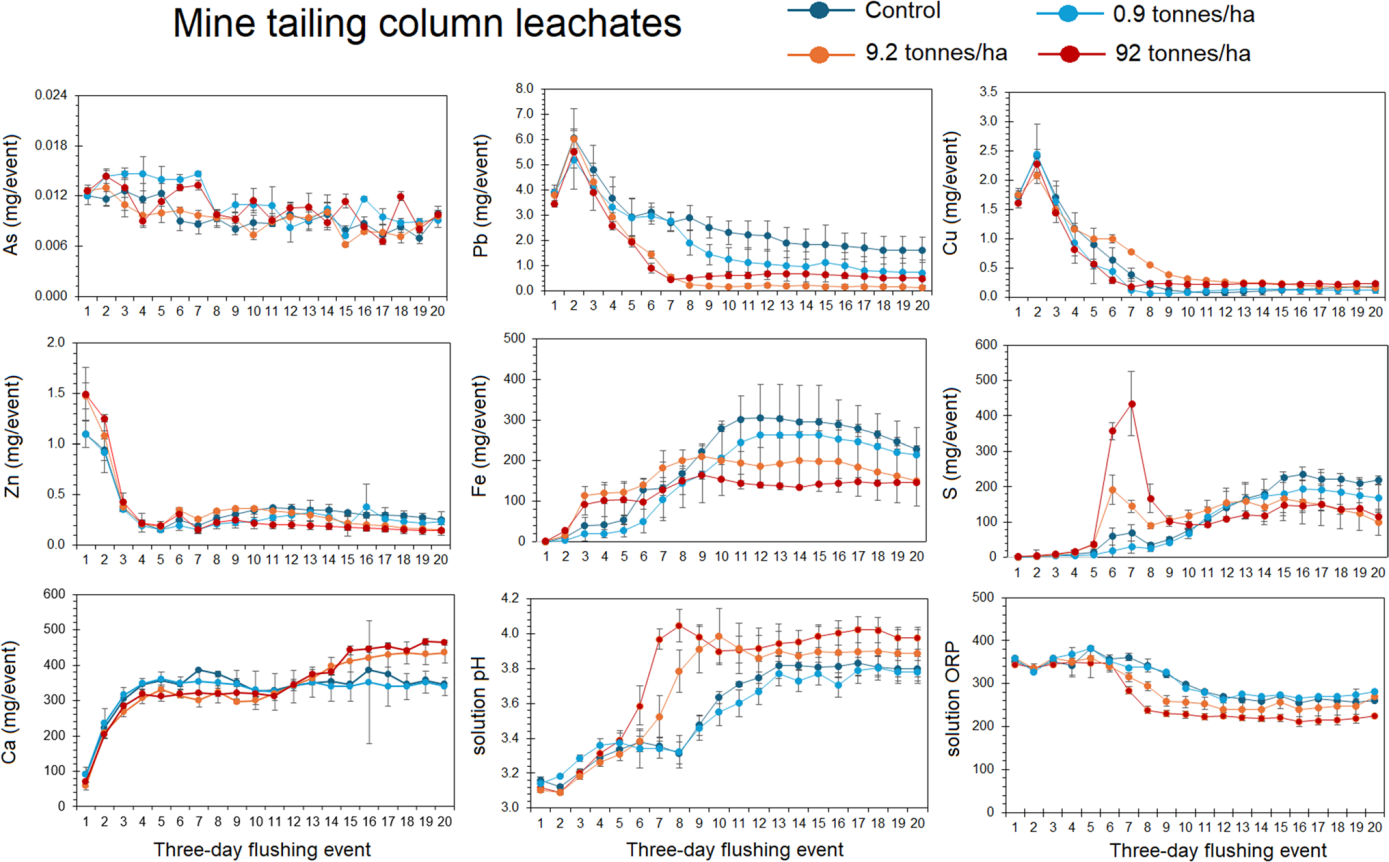
Mine tailing column experiment leachate elemental masses and chemical properties. Each column containing 1.5 kg of mine tailings from Jenkins Mine (JEN) was treated with one of four treatments: control, biochar and lime 0.92 tonnes/ha, biochar and lime 9.2 tonnes/ha, and biochar and lime 92 tonnes/ha. These treatments were applied on the 5th 3-day flushing event. Every three days, 360 mL of 10 mM NaCl and 10 mM CaCl2 solution was applied to each column for 20 events over a 60-day period. Error bars and standard error and *n* = 3 for each treatment

Our column experiment to evaluate increasing application rates of combined limes and biochar to increase pH and adsorb PTEs from Jenkins Mine (JEN) mine tailings found that application of biochar and lime can reduce certain PTEs and somewhat improve pH (Fig. 9). Our results show that increasing biochar and lime application significantly decreased the mass of Pb, Zn, and Fe in leachate as well as increased final leachate to pH 3.9 for 9.2 tonnes/ha application rate and pH 4.0 for 92 tonnes/ha application rate (Fig. 9). The biochar and lime increased adsorption of metals through sorption to charge sites and oxygen functional groups and neutralized acidity through dissolution of CaCO_3_ and CaO promoting precipitation (Oh and Yoon 2013; Novak et al. 2018). Significantly higher leachate Ca for the 9.2 and 92 tonnes/ha treatments confirm the dissolution of Ca minerals. The decreased leaching of Fe can be ascribed to the increase in solution pH promoting precipitation of Fe oxyhydroxides and adsorption of Pb and Zn to their charged sites (Jönsson et al. 2006; Ying et al. 2022). Increasing treatment rates did not significantly decrease mass of As and Cu in leachate. We expected the higher pH and formation of Fe oxyhydroxides to promote As and Cu sorption, but our results suggest this did not occur. Sun et al. (2022) showed that wood biochar without chemical modifications have low As removal rates < 40% of added As^+5^ between pH 3 and 5 due to competition by phosphate and carbonate ions as well as less surface charges and complexation by oxygen functional groups on biochar due to the low pH (< 4) (see Niazi et al. 2018; Sharma et al. 2022). Considering Cu, Tomczyk et al. (2019) found adsorption of Cu by biochar to be strongly pH dependent of the solution and dependent on the surface area and surface charge. Column leachate ORP was also significantly lower for the 9.2 and 92 tonnes/ha treatments than the control and 0.9 tonnes/ha treatments, which may also effect surface charges and complexation by biochar. Lastly, we observed a spike in S for the 9.2 and 92 tonnes/ha followed by significantly lower S for the 9.2 and 92 tonnes/ha. We hypothesize that the initial flush of S was sulfate desorption and mobilization from the mine tailings from carbonate and bicarbonate from the lime. These results show the biochar and lime can significantly decrease the mobility of certain PTEs such as Pb and Zn, increase leachate pH, and increase leachate Ca. However, biochar and lime were not effective for decreasing As and Cu as their concentrations did not significantly decrease. Moreover, our experiment showed that at least 9 tonnes/ha would be needed for a significant effect and our experimental design assumed treatment of only the top 30 cm of the mine tailings. Additional biochar and lime would not generate significantly lower reduction in Pb and Zn or pH necessarily. If the mass of mine tailings to be treated is much deeper, then the required application rate could be much greater than 9 tonnes/ha. Acharya and Kharel (2020) discuss that active treatments of AMD through liming are expensive either through the acquisition of the ash or lime or the prolonged required use to effectively quench the AMD. Passive treatments not evaluated here such as industrial byproducts and bacterial augmentation may also be effective, but without an effective understanding of the AMD source, these options cannot be recommended for implementation at the studied mines and their tailings.

## Conclusions

The overarching goal of this study was to quantify current potentially toxic elements (PTEs) and nutrient pollution across tributaries into Lake Anna and their relationship to former mines and agricultural lands as well as physicochemical properties and processes governing their transport. We found limited enrichment of P in stream waters which was associated with DOM, Fe, but not agricultural land cover across subwatersheds. Both suspended sediments and bottom sediment P concentrations were above concentrations that have been shown to cause eutrophication but were weakly associated with %OM, agricultural land-use, or clay sized particles. Particulate transport of P in streams was more important than dissolved transport across the subwatersheds. By comparing the subwatershed export and the Lake Anna outflow export, Lake Anna is a sink for dissolved P (79% retention) and suspended sediment P (56% retention) but still releasing over 0.55 Mg/year dissolved P and 107 Mg/year suspended sediment P to the Lake Anna and potentially to the Chesapeake Bay.

Stream water, suspended sediments, and bottom sediments across the subwatersheds show that Pb and Cu concentrations, and to a lesser extent Cr and Zn concentrations, were enriched above levels considered ecologically hazardous consistently at Contrary Creek (CC). Total annual export and area normalized annual export highlight that CC was a dominant source of PTEs (specifically Cu, Pb, and Zn) to the Lake Anna watershed. Particulate transport was found to be more important than dissolved transport across the Lake Anna watershed.

For our second objective, we evaluated if exposed mine tailings can be further reclaimed using biochar and lime. Surface samples from AS, CP, and NLP mine tailings showed hazardous levels of As and Pb enrichment but limited bioavailability based upon pine needle PTE concentrations. Our mine tailing column experiment used exposed mine tailings from the Jenkins Mine to evaluate further reduction in mobile phases of PTEs using biochar and lime. Our findings show that application of 9.2 tonnes/ha of lime and biochar could substantially diminish the leaching of Pb and Zn and increase leachate pH but could not significantly reduce As or Cu from the mine wastes. Additional research on subsurface transport pathways and distribution/mobility of legacy pollution is warranted, as findings from our study imply both sources are important steps to reducing PTE loading to Lake Anna.

## Data Availability

Stream water, bottom sediment, and suspended sediment data are available as CSVs on the University of Virginia Dataverse: https://dataverse.lib.virginia.edu/.
